# Promoting the Mathematization of Physics: Hermann Minkowski's Draft Presentations of His 1908 “Grundgleichungen” Paper

**DOI:** 10.1002/bewi.70018

**Published:** 2026-05-11

**Authors:** Tilman Sauer

**Affiliations:** ^1^ Institut für Mathematik Johannes Gutenberg‐Universität Mainz Mainz Germany

**Keywords:** Einstein, history of special relativity, manuscripts, mathematization, Minkowski

## Abstract

I present and discuss two drafts of remarks prepared by the mathematician Hermann Minkowski (1864–1909). They were composed in December 1907 while preparing his paper on the “Basic Equations of Electromagnetic Processes in Moving Bodies” for publication in the Proceedings of the Göttingen Academy of Sciences. I discuss the biographical and institutional context as well as the scientific background to highlight a document which illuminates the perception of an emerging academic discipline of mathematical physics.

## 1 Introduction

Hermann Minkowski (1864–1909) was a well‐known Göttingen mathematician and prominent contributor to the early development of the theory of relativity.^1^ He is credited with the introduction of a manifestly four‐dimensional conception and mathematical representation of special relativistic spacetime.^2^ Due to his untimely and unexpected death on January 12, 1909, from a ruptured appendix, he only published a single paper on relativistic electrodynamics, a paper entitled “Basic Equations of Electromagnetic Processes in Moving Bodies” (“Grundgleichungen für die elektromagnetischen Vorgänge in bewegten Körpern”).^3^ A famous talk on “Space and Time” (“Raum und Zeit”) in which he presented his vision of four‐dimensional, later so‐called “Minkowski spacetime” to the Cologne meeting of the Society of German Natural Scientists and Physicians (Gesellschaft Deutscher Naturforscher und Ärzte, GDNÄ) was prepared by him for print but appeared only after his death.^4^

In this paper, I present and discuss two draft versions of remarks in which Minkowski characterized the content of his “Grundgleichungen” paper from 1908. The drafts are obviously Minkowski's prepared remarks addressed to his Academy colleagues at the occasion of his submission of the article at a regular meeting of the Göttingen Society of Sciences (Göttinger Gesellschaft der Wissenschaften, GGW) for publication in its Proceedings (*Nachrichten*) on December 21, 1907, which is the official date of submission of the paper. Both versions, a manuscript and a typescript, display many emendations which show Minkowski's intention to find the right phrases for a presentation of his paper, and they can also be read as an early version of the introductory section of the published paper.^5^ As such they are of interest for a critical genetic analysis of mathematical texts.^6^ They reveal how Minkowski advocated his own academic discipline toward his colleagues.^7^ Minkowski's presentation of his technical work was addressed to the full spectrum of established academics and, in particular, the alterations, deletions, and rephrasing of the text reveal his perception and deliberations of placing his own work located in the intersection of mathematics and physics into a broader disciplinary setting. Further interest in these drafts derive from the fact that they reveal an unusual insight into the Academy's publication practice of the time.

## 2 Biographical and Institutional Context

When Minkowski became full professor of mathematics in Göttingen in 1902, he was a young but already well‐established mathematician. He had received early fame as recipient of the *Grand prix des sciences mathématiques* of the French Academy of Sciences in 1883, while still a student in Königsberg, for a manuscript on the theory of quadratic forms.^8^ Before coming to Göttingen, he had taught at the universities of Bonn and Königsberg as well as at the Eidgenössische Polytechnicum (today's ETH) in Zurich, where he was one of Einstein's mathematics teachers. His appointment to Göttingen was promoted by Felix Klein (1849–1925) and David Hilbert (1862–1943) in consonance with Friedrich Althoff's (1839–1908) plans to establish Göttingen as a center for mathematics and physics. Prior to his arrival in Göttingen, Minkowski had become a corresponding member of the mathematical‐physical class of the GGW in 1901.^9^

Minkowski's decision to publish a long and technical paper on the basic equations of electrodynamics in the Proceedings of the GGW rather than, say, in the *Mathematische Annalen* or the *Annalen der Physik* may have been motivated by various deliberations. Among those may have been concerns about his institutional standing among his colleagues in Göttingen, and also its interdisciplinary nature between mathematics and physics.

Minkowski had published in the *Nachrichten* before. Three papers of his had been presented for publication by Hilbert,^10^ and after being elected corresponding member of the Academy, he had presented another number‐theoretic paper himself which was duly published as a regular article.^11^ Yet another paper of his was presented by Minkowski in the session of July 8, 1905. As the *Geschäftliche Mittheilungen* of that meeting indicate it was entitled “Über automorphe Funktionen” and was said to be appearing in the *Nachrichten* ^12^ but no such paper by Minkowski was ever published there. Minkowski had also presented someone else's paper^13^ for publication in the session of January 26, 1907.

Despite these academic activities of Minkowski, the large majority of mathematical papers printed in the *Nachrichten* in those years were presented by either Klein or Hilbert.

In March 1908, the list of members of the Göttingen Academy comprised 28 full members (“ordentliche Mitglieder”), 14 each in the mathematical‐physical and in the philological‐historical class, a fixed number. Of those full members only the astronomer Karl Schwarzschild (1873–1916) was younger than Minkowski. The oldest were the zoologist and comparative anatomist Ernst Ehlers (1835–1925), who was also one of the two secretaries, and the legal scholar and historian Ferdinand Frensdorff (1833–1931). All full members were also full professors of the Göttingen university faculty and about half of them also carried the additional title of Privy Council (“Geheimer Regierungsrath”).^14^ In addition, the Academy comprised 43 external members, and some 93 corresponding members in the mathematical‐physical class as well as 82 corresponding members in the philological‐historical class.

Minkowski's affiliation with the Society as a corresponding member while being a Göttingen resident was slightly unusual. Of all corresponding members, only Minkowski and his mathematician colleague Carl Runge (1856–1927) were listed as residents of Göttingen.^15^ Like Minkowski, Runge was full professor in Göttingen, after a fourth chair for mathematics had been created in 1904 with the intent of establishing Göttingen as the academic center of mathematics by Althoff. Minkowski's and Runge's affiliation with the Academy reflected the extraordinary situation of mathematics in Göttingen, which was then equipped with the unusually large number of four chairs in mathematics. Both mathematicians were recently appointed full professors and had been elected corresponding members in 1901 but had to wait for a vacancy before they, too, could be elected full members. Runge was elected full member to the GGW in 1914.

## 3 Scientific Context

When Minkowski decided to present his paper on the foundational equations of electrodynamics of moving media for publication to the Academy he was sufficiently confident that his work was worthy of publication by that institution even though at that date the manuscript in all likelihood was not yet completed.

Minkowski had been good friends with the mathematicians David Hilbert and Adolf Hurwitz (1859–1919) since his student days in Königsberg,^16^ and when he and Hilbert had become colleagues in Göttingen they began to pursue a common interest in studying recent physics. Their aim was to analyze advanced physical theories from a mathematical point of view, as Hilbert had proclaimed prominently in the sixth of his famous “Mathematical Problems”^17^ for the twentieth century.^18^ In 1905, they had conducted a seminar, jointly with Emil Wiechert (1861–1928) and Gustav Herglotz (1881–1953), on electron theory, in which they studied works by Hendrik Antoon Lorentz (1853–1928), Henri Poincaré (1854–1912), Heinrich Hertz (1857–1894), Max Abraham (1875–1922), and others.^19^

Einstein's paper on “Electrodynamics of Moving Bodies,”^20^ which introduced Einstein's special theory of relativity, only came out in September 1905 and was not included in the seminar syllabus. But in a letter to Einstein dated October 9, 1907, Minkowski asked for offprints of this paper.^21^ A month later, Minkowski gave a presentation to the Göttingen Mathematical Society (Göttinger Mathematische Gesellschaft, GMG), a circle of mathematicians founded by Felix Klein and Heinrich Weber (1842–1913) in 1892^22^ which met regularly during semesters and discussed recent literature as well as recent work of the participants. Minkowski's presentation at this event was posthumously published on the basis of a typescript of the talk in an edited version by Arnold Sommerfeld (1868–1951) under the title “Das Relativitätsprinzip.” It was, in fact, published twice, both in a journal read by physicists (*Annalen der Physik*) and in one read by mathematicians (*Jahresbericht der Deutschen Mathematiker‐Vereinigung*, *JDMV*).^23^ The abstract of this talk delivered to the GMG on November 5, 1907, is given in the “Mitteilungen und Nachrichten” section of the *JDMV* as follows:

H. Minkowski develops a new form of the electrodynamic equations following the new investigations into the principle of relativity in electrodynamics. He sets time equal to a fourth space coordinate, except for a purely imaginary factor, and combines the electrical and magnetic quantities into four‐dimensional vectors, also adding suitable imaginary factors. This gives the equations a completely symmetrical form, which remains invariant under all orthogonal transformations of the four‐dimensional space. If the general principle of relativity is maintained for matter in motion, a new form of the electrodynamic equations results for this case as well, if it is noted that every state of motion is transformed into a state of rest by a suitable transformation of the same kind, provided that only the transformed fourth variable is again regarded as imaginary time. Finally, the lecturer suggests applications of the principle of relativity to dynamics and to the theory of gravitation.^24^

At this point, Minkowski must already have made substantial progress in writing up his 1908 paper. Six weeks later, on December 10 in another session of the GMG, Felix Klein spoke about quaternion theory, and the abstract in the “Mitteilungen und Nachrichten” notes that

Subsequently, H. Minkowski (cf. the lecture of November 5) shows how the equations of electrodynamics can be simplified even further if the electric and magnetic quantities are combined into vectors or quaternions with complex components (biquaternions). The basic equations of electron theory can then be summarized in a single biquaternion equation of the simplest kind, and the most general equations resulting from the principle of relativity for moving matter can also be written very simply.^25^

Another 10 days later, Minkowski presented his work on the *Grundgleichungen* to his colleagues at a regular session of the GGW for presentation in its *Nachrichten*. It might be observed that the printed version mentioned neither quaternions nor biquaternions.

## 4 Discussion of the Drafts

Two draft versions of remarks prepared by Minkowski for presentation of his paper to his Academy peers are preserved in the Archives.^26^ One version is a manuscript, the other a typescript, both of them were heavily emended, and both versions bear some resemblance to the introductory section of his published paper.

Minkowski begins by placing his own work into a historical problem setting which he defines by the names and recent work of three physicists, Heinrich Hertz, Hendrik Antoon Lorentz, and Albert Einstein.

Minkowski knew Hertz's work well from his time in Bonn. Minkowski had been in Bonn from 1887–1894, and Hertz had been professor of physics there from 1889 until his death on January 1, 1894.

He begins by referencing Hertz's work “Ueber die Grundgleichungen der Electrodynamik für ruhende Körper.” That paper had a history with the Academy. It was published twice. It first appeared in the *Annalen der Physik und Chemie* in 1890.^27^ The byline of that paper states that it had been “Aus den Gött. Nachr. […]; mitgetheilt vom Hrn. Verf.” Indeed, the very same paper was also printed in the *Nachrichten* that year.^28^ It had been presented to the GGW in the session of March 1, 1890. Its byline states: “Nachträglich vorgelegt von Voigt.” In 1908, Woldemar Voigt was still a member of the Academy. In the printed version of his “Grundgleichungen” paper, Minkowski, however, cites only the *Annalen* version and the reprint in Hertz's *Untersuchungen ueber die Ausbreitung der elektrischen Kraft*.^29^

This first paragraph is fairly similar in both draft versions and in the published version. Minkowski praises Hertz's work in electrodynamics in various formulations as foundational (“grundlegend”), or fundamental (“fundamental”), or perfected (“vollendet”). For the basic equations for bodies at rest, it is characterized as no longer controversial, whereas his equations for moving bodies would have been not (yet), fully or in all its consequences, confirmed by experiment. The first paragraph in the published version also begins with a characterization of Hertz's work. But it is more distanced, commenting only on Hertz's approach to the problem of moving bodies, which Minkowski says had to be given up due to adverse experimental results.

The second paragraph in both draft presentations seeks to find a characterization of Lorentz's work in general. Lorentz had been elected external member to the GGW only recently in 1906. Minkowski calls his work new, having succeeded in producing excellent (“grossartige”) or great (“grosse”) insights. But his theory, he suggested, was marred by many complicated or hypothetical conceptions and it could only be seen as a temporary step in a path toward more perfected or satisfactory or definitive laws. Again this paragraph is mirrored in the published version which says that Lorentz's theory appears to be justified in its bold hypotheses by its great successes.

The third paragraph in the draft presentations introduces both Lorentz's contraction hypothesis and Einstein's most recent work. The contraction hypothesis is Minkowski's point of departure, and he struggles, here as in several other manuscripts, to arrive at a proper characterization of this hypothesis. Here he calls it very complicated (“sehr kompliziert”), or highly curious (“höchst verwunderlich”), or strange‐sounding (“seltsam klingend[]” or “anmutend[]”). In any case, Einstein's interpretation of this hypothesis, in Minkowski's understanding, is the starting point for Minkowski's own work. Einstein here is introduced first as a young Swiss physicist, then as a physicist of German descent (“von Hause aus Deutscher”), now active in Bern. In the typed version both references to Einstein's nationality were dropped and only his current placement in Bern is kept. In any case, Einstein, in Minkowski's understanding, has somehow transformed Lorentz's contraction hypothesis into the relativity principle. Again, Minkowski is searching for the right phrases. He first tries saying that Einstein would have “peeled out” (“herausgeschält”) of that hypothesis a particularly simple and, he adds, clear core. The typed version first tries a very different phrase. Einstein would have extracted from the contraction hypothesis a simple and extremely tasty (“einfachen und höchst leckeren”) core, then he corrects to a simply clear and nutritious (“einfach klar und gehaltvoll”) core, and finally settles with simply clear and meaningful (“bedeutungsvoll”).

It is at this point that the draft presentations begin to differ substantially from the published version. There, he first explains Lorentz's theory in a more detailed manner, then mentions Poincaré, whom he associates with the proper recognition of the Lorentz transformations, and then juxtaposes what he calls a theorem of relativity, a postulate of relativity, and a principle of relativity. According to Minkowski, Lorentz had established as a theorem the mathematical fact that the electromagnetic equations are covariant under Lorentz transformations. Lorentz also expressed the postulate that such covariance shall further be valid for laws which are not yet known, specifically by formulating his contraction hypothesis. Minkowski credits Einstein with having expressed most sharply (“am schärfsten zum Ausdruck gebracht”) that the postulate of relativity was not simply an artificial hypothesis but a new conception of the notion of time required by the phenomena (“eine durch die Erscheinungen sich aufzwingende neuartige Auffassung des Zeitbegriffs”). As for the principle of relativity, it would not yet have been fully formulated in all generality. By formulating this principle, Minkowski intends to show that the electromagnetic equations are uniquely determined in a way that is different from all prior formulations.

The fact that the draft versions for the presentation are both rather similar but diverge substantially from the published version shows that Minkowski may have intended the presentation as draft introductions but he still went a long way before arriving at the final published words.

In any case, in both draft versions, Minkowski emphasized that the relativity principle, or rather this new law of inertia, as he calls it, would, if found to be true, “revolutionize our prior concepts of space and time” (“unsere bisherigen Vorstellungen von Raum und Zeit umwälzen”). It is then interesting to see how Minkowski tries to find words to justify the esoteric mathematical character of his work. He emphasized that the mathematical content of his paper would be straightforward to understand for mathematicians with a training in recent current developments and methods. On the other hand, it would be less accessible for physicists. Clearly, a dilemma arises. While the mathematical language is needed for an explication of the physical ideas, it also renders the work inaccessible to non‐mathematicians. Minkowski very pointedly highlights this difficulty. If he were to explain the content of his paper in proper mathematical terms to his colleagues, he would appear to them as an “Übermensch” from the psychiatric clinic of “His Magnificence.” The latter expression is the old address for the rector of Göttingen University, at the time August Cramer (1860–1912). Cramer held the post of rector in the academic year 1907/08^30^ and was a well‐known psychiatrist, full professor at Göttingen, founding director of the first fully state‐funded sanatorium for treatment of psychiatric conditions, the so‐called “Rasemühle.” Cramer was not a member of the GGW. But the allusion was clear: Minkowski did not even try to present details of his paper in mathematical terms, lest he would appear as a lunatic.

Nevertheless, Minkowski did try to give the esoteric mathematics of his work a twist of mystery. One of the innovations of his paper was the introduction of an imaginary time coordinate. In mathematical terms this amounted to a multiplication of the time coordinate by a factor of $i=\sqrt{-1}$, the imaginary unit. This formal manipulation allowed him to rewrite the set of the three spatial coordinates *x*, *y*, *z*, and the time coordinate *t* as four coordinates *x*_1_, *x*_2_, *x*_3_, *x*_4_, with *x*_4_ = *ict*, where *c* is the speed of light. The change of notation allowed a mathematical formulation which manifestly expressed the equivalence of the space and time coordinates in the four‐dimensional setting. The Lorentz transformations of special relativity can then be represented in a way which reveals a formal similarity to simple rotations in space. For a description of rotations in space, concepts and tools of trigonometry are used, and these now appear in very similar terms also for the formal description of relativistic spacetime transformations. Minkowski then alluded to Leonhard Euler's famous relation expressing the trigonometric functions of sin(*x*) and cos(*x*) in a unified framework in complex analysis as real and imaginary parts of Euler's exponential function, exp(*ix*) = cos(*x*) + *i* sin(*x*). In this framework, the imaginary unit *i* can be associated with a rotation angle in ordinary space of exactly 90^o^ or, equivalently *π*/2 radians, since exp(*iπ*/2) = *i*. Here *π* is, of course, the circle number, i.e., the ratio of a circle's circumference to its diameter. Minkowski refers to it by the old epithet of “Ludolph's number.” Since the formal introduction of an imaginary time coordinate *x*_4_ = *ict* also involves the speed of light *c* with a numerical value of 3·10^10^ cm/sec, it also changes the physical dimension of the time coordinate. Minkowski now tried to capture the essence of this formal innovation like this:

thus, according to the new relativity principle, 1 cm is to the time of 1/30000 of a millionth of a second as two right angles is to Ludolph's number *π*.^31^

Minkowski labors on a pointed formulation of this equivalence in both draft versions but only to add immediately that he does not expect any non‐mathematician to be able to make any sense of it.

None of these witty comments made it into the introduction to his published paper. It is only in the final paragraphs of his draft presentation that Minkowski gives a brief characterization of what his paper is actually about. In doing so, he is not overly modest. He announces a new system of equations for electrodynamics and expresses his confidence that with these new equations the true foundations of electrodynamics have indeed been found.

## 5 Concluding Remarks

Almost immediately after Minkowski's paper was published in the *Nachrichten*, Einstein expressed doubts about the physical validity of Minkowski's version of the relativistic form of the electromagnetic field equations in the presence of matter. The topic, in fact, remained controversial for quite some time, and it would have been interesting to see how Minkowski would have reacted to the later debate. He had already been working on a sequel paper to his 1908 paper, a version of which was prepared for print by Max Born and posthumously appeared in 1910 in the *Mathematische Annalen* and was also included in his *Collected Works*.^32^ But Minkowski's sudden death prevented his active participation in the later history of relativistic electrodynamics or in the general theory of relativity for that matter. His fame as the originator of a concept of “Minkowski spacetime” largely arose from his “Raum und Zeit” talk that he gave shortly before his death to the annual Assembly of German Scientists and Physicians (Versammlung Deutscher Naturforscher und Ärzte) in Cologne in September 1908.^33^ The published version^34^ of that talk was a carefully crafted text, which did not fail to exert a lasting influence in the history of modern physics. Minkowski was quite conscious of the role of language in promoting his ideas and in promoting science in general. In manuscript notes for a talk addressed to mathematics and physics teachers,^35^ he explicitly said that he would use “more diplomatic language” in his publications but would speak more freely when addressing them directly. In the draft presentations to his peers at the Göttingen Academy he can be seen trying to find the right words to promote a mathematization of science to an established institution of academic scholarship.

## 6 Appendix: Drafts

### 6.1 Manuscript

Am 19.März 1890 wurde für die ⌊gab Heinrich Hertz in den⌋ Gött.Nachr. die Arbeit von Heinrich Hertz eine grundlegende ⌊fundamentale⌋ Darstellung der Maxwellschen Theorie und seitdem stehen die ⌊Grund⌋Gleichungen für^36^ die elektromagnetischen Vorgänge in ruhenden Körpern in einer bestimmten ⌊nicht mehr angefochtenen⌋ Weise fest. Dagegen haben ⌊sind⌋ die Ansätze, welche Hertz kurz danach für die Elektrodynamik bewegter Körper gab, noch nicht an durch die Erfahrung vollauf bestätigt worden. 1895 entwickelte ⌊H.A.⌋ Lorentz auf Grund atomistischer Vorstellungen ⌊der Elektrizität⌋ eine neue Theorie der elektrischen und optischen Erscheinungen von ⌊in⌋ bewegten Körpern, die zu grossartigen Erfolge zeitigte. Immerhin war diese ⌊Grundlagen dieser⌋ Theorie noch mit soviel ⌊derart von komplizierten⌋ hypothetischen Vorstellungen durchsetzt, dass man ihr ⌊sie⌋ ⌊in ihr⌋ immer nicht mehr als ⌊nur erst⌋ einen provisorischen Durchgang zu vollkommeneren befriedigenderen Gesetzen sehen möchte. Aus zum Teil sehr komplizierten höchst verwunderlich ⌊und seltsam anmutenden⌋ klingenden Voraussetzungen der Lorentzschen Theorie, es sollen sich alle ⌊jeder⌋ Körper der in Bewegung gerät, eine bestimmte Kompression in Richtung seiner Bewegung erfahren, hat vor zwei Jahren ein junger Schweizer Physiker, ⌊der jetzt in Bern wirkt⌋ A. Einstein der jetzt in Bern wirkt, von Hause aus ein Deutscher ist, einen besonders ⌊klaren und⌋ einfachen Kern herausgeschält, das von ihm sogenannte Relativitätsprinzip. Es ist das ein Gesetz, das aus ⌊an⌋ die Stelle des Galileischen Trägheitsgesetzes zunächst in der Elektrodynamik ⌊bei⌋ elektrodynamischen Vorgängen und dann unzweifelhaft in der ganzen Physik treten würde. Dies Gesetz würde ⌊nun⌋ unsere wenn es sich wirklich ⌊ganz⌋ bewahrheiten wird, woran zu glauben ⌊was zu hoffenanzunehmen⌋ man gute Gründe hatte, bisherigen Vorstellungen von Raum und Zeit ganzlich umwälzen. Es würde schwer sein, ohne ⌊fällt⌋ ist für den Mathematiker ⌊bei der Entwicklung, welche die reine⌋ Mathematik im letzten Jahrhundert genommen wird, für ⌊fällt⌋ dem Mathematiker selbst recht ⌊besonders⌋ leicht, leichter als den Physikern, die ⌊genaue⌋ Tragweite dieses Gesetzes ⌊neue Trägheitsgesetz⌋ zu verstehen und sich dafür zu enthusiasmieren.

[*in the left hand margin*: weil es in der Tat mathematisch so also viel befriedigender als das Galileische Trägheitsgesetz ist]

Immerhin würde es recht schwer sein, ohne spezielle mathematische Vorkenntnisse ⌊und es ist auch nicht meine Absicht dies ––?⌋ dieses ⌊neue Trägheits⌋Gesetz ohne spezielle mathematische Ausführungen klar zu machen. Ich könnte nur höchstens mit scheinbar verständlichen Worten Sätze so formulieren, die in der Tat das Wesen der Sache, wie ein Mathematiker zugeben würde, im Innersten treffen, für einen Unkundigen aber doch nur wie Aussprüche von ⌊eines⌋ Übermenschen, sagen wir aus der Anstalt ⌊Klinik⌋ seiner Magnifizenz klingen würden.

[*in the margin*: Es wird durch das [––] die genaue Beziehung zwischen Zeit und Länge mit Rücksicht auf die Fortpflanzungsgeschwindigkeit des Lichtes gewahrt]

Z.B. das Resultat indem ich auf die ⌊Eulers⌋ grosse Entdeckung vom Zusammenhang der trigonometrischen Funktionen mit den Logarithmen Bezug nehme, welche seitdem die Entwickelung der Mathematik völlig beherrscht, so ist ⌊wird manche eine Beziehung zwischen Zeit und Länge verknüpft festgestellt, und ⌊zwar⌋ verhält sich⌋, nach dem neuen Relativitätsprinzip 1 cm zu der Zeit ⌊die Zeit annähernd⌋ 1/30000 von einem millionsten einer Sekunde ⌊zu einem 1 cm⌋ wie 2 rechte Winkel zu der Ludolphschen Zahl *π*. ⌊Ich verlange nicht, dass dieses so begriffen werde, aber⌋ Wenn ich Sie versichere, dass dieser Behauptung ein ganz bestimmter Sinn ganz bestimmte experimentelle Tatsachen einen sehr vernünftigen Sinn verschaffen, so werden Sie, wenn Sie es mir glauben wollen, jedenfalls überzeugt sein, dass es mit dem Relativitätsprinzip eine höchst merkwürdige Bewandtnis hat. Genug, das Relativitätsprinzip behauptet ⌊kommt⌋ in ganz bestimmten Sinne zu dem Resultate ⌊Schluss⌋, dass dem Eigenschaften ⌊Zustand⌋ Ruhe gegenüber der Bewegung ⌊durchaus? gar⌋ keine⌊rlei⌋ Eigenschaft der Erscheinungen entsprechen.

Ich habe mir nun die Frage vorgelegt, ob denn die Lorentzsche Elektrodynamik bewegter Körper, aus welcher sich das Relativitätsprinzip herausgeschält hat, mit der ⌊schliesslichen⌋ Forderung des Relativitätsprinzips in tatsächlich im Einklang steht, und da bin ich zu merkwürdigen Resultaten gelangt. Es ist dies wohl bis zu einem gewissen Grade durchaus ⌊nicht⌋ vollständig, aber bis zu eine gewissen Grade, nämlich für unmagnetisierbare Körper, aber ⌊auch⌋ hier nur durch wegen eines Zufalls, nämlich weil Lorentz die Forderung ⌊Bedingung⌋ des Nichtvorhandenseins von Magnetismus gerade in einer solchen Weise eingeführt hat, die ihrerseits durchaus nicht dem Relativitätsprinzip entspricht, also ⌊Damit also die Lorentzsche ⌊Bedingung⌋ dem Relativitätsprinzip entspreche, muss sie⌋ gewissermassen rein zufällig durch eine Kompensation von zwei Widersprüchen. Andererseits ⌊––⌋ ist die Art, wie Lorentz den ⌊elektrischen⌋ Strom in der sind die Beziehungen, welche Lorentz zwischen elektrischem Strom ⌊und⌋ elektromotorischer Kraft mittelst der Leitfähigkeit und zwischen elektrischer Erregung Verschiebung und elektromotorischer Kraft mittelst der Dielektrizitätskonstanten ansetzt, ebenfalls nicht ⌊––?⌋ mit dem Relativitätsprinzip im Einklang. Ich habe nun weiter ein rechtes System von Gleichungen für die Elektrodynamik bewegter Körper aufgestellt, ⌊und das Gesetz das ⌊betreffende⌋ System ist durch die Forderung völlig bestimmt⌋, welche in allen Hinsichten dem Relativitätsprinzip entspricht und ich glaube auch die Hoffnung hegen zu dürfen ⌊bringe gute Gründe dafür bei⌋, dass mit diesen neuen Gleichungen ⌊in der Tat damit die wahren⌋ Grundlagen für die Elektrodynamik bewegter Körper gefunden sein mögen. Meine gegenwärtige Darstellung sucht mit einem Minimum von mathematischen Ausführungen durchzukommen, indem sie sich in erster Linie an die Physiker wendet. In einem späteren Ein zweiter Aufsatz, den ich nach den Ferien vorlegen will, soll speziell ⌊mehr⌋ mathematisch Beiträge zur Elektrodynamik bringen. Der ⌊gegenwärtige⌋ Aufsatz ist etwa 1 Bogen stark und ich bitte ihn in die Nachrichten aufzunehmen.

### 6.2 TYPESCRIPT

1890 gab Heinrich HERTZ in den Göttinger Nachrichten eine grundlegende vollendete Darstellung der MAXWELLschen Theorie und seit jener Arbeit stehen die Grundgleichungen für die elektromagnetischen Vorgänge in ruhenden Körpern in ⌊einer⌋ nicht mehr angefochtenen Weise fest. Dagegen sind die Ansätze, die HERTZ kurz danach für die Elektrodynamik bewegter Körper gab, durch die Erfahrung nicht voll ⌊in allen Konsequenzen⌋ bestätigt worden. 1895 entwickelte ⌊dann⌋ H.A. LORENTZ auf Grund atomistischer Vorstellungen der Elektrizität eine Theorie der elektrischen und optischen Erscheinungen ⌊in bewegten Körpern⌋, die ;grossartige ⌊grosse⌋ Erfolge zeitigte. Die Grundlagen dieser Theorie sind aber noch derart von komplizierten ⌊kühnen⌋ hypothetischen Annahmen durchsetzt, dass man in ihr ⌊in den Lorentzschen Ideen⌋ nur einen provisorischen Durchgang zu den definitiven Gesetzen sehen möchte. Aus den zum Teil höchst seltsam anmutenden Aussagen ⌊Voraussetzungen⌋ der LORENTZschen Theorie ⌊–⌋ es soll jeder ⌊im leeren Raume⌋ bewegte Körper Kompressionen in Richtung ⌊Veränderungen seiner Dimensionen nur allein wegen⌋ seiner Bewegung erfahren⌊–⌋ aus diesen höchst verwunderlichen Annahmen, welche ersonnen sind, um ⌊a priori⌋ die experimentelle Unmöglichkeit ⌊darzutun⌋, eine Relativbewegung der Erde gegen den Lichtäther wahrzunehmen, a priori zu begründen ⌊aus diesen Hypothesen⌋, hat vor zwei Jahren ein junger Physiker, A. EINSTEIN in Bern, einen einfachen und höchst leckeren ⌊einfach klaren und höchst gehaltvollen bedeutungsvollen⌋ Kern herausgeschält, das von ihm sogenannte Relativitätsprinzip. Es ist das ein Gesetz, das an die Stelle des Galileischen Trägheitsgesetzes zunächst bei elektromagnetischen Vorgängen und dann unzweifelhaft im ganzen Umkreis der Physik treten würde. Dieses neue Trägheitsgesetz würde, wenn es sich voll bewahrheiten wird, was zu hoffen man gute Gründe hat, unsere bisherigen Vorstellungen von Raum und Zeit total umwälzen. ⌊–⌋ Bei der⌊jenigen⌋ Entwicklung, welche die reine Mathematik im letzten Jahrhundert genommen hat, fällt es dem reinen Mathematiker besonders leicht, leichter als dem ⌊einem dem modernen⌋ Physiker, sich in das neue Gesetz hineinzudenken und sich dafür zu enthusiasmieren, weil es in der Tat mathematisch sich sehr viel ⌊theoretisch sich in vielen Hinsichten viel⌋ befriedigender ⌊anlässt⌋ als das Galileische Gesetz formuliert. Es würde freilich nicht leicht fallen, und ich werde es deshalb jetzt ⌊in diesem Momente⌋ auch gar nicht versuchen, dieses neue Trägheitsgesetz vor Nichtmathematikern auch nur in Worte zu kleiden. Ich könnte höchstens ⌊allenfalls⌋ mit scheinbar verständlichen Worten einige Sätze fassen ⌊formulieren⌋, die in der Tat, wie ein Mathematiker zugeben müsste, das Wesen der Sache im Innersten treffen, die aber für einen Unkundigen nur mehr wie Aussprüche, sagen wir eines Übermenschen ⌊sagen wir nun aus⌋ der ⌊einer psychiatrischen⌋ Klinik der derzeitigen Magnifizenz ⌊unseres her⌋ klingen ⌊klingen tönen⌋ ⌊möchten⌋.⌊––⌋ Es wird durch das Relativitätsprinzip eine gewisse Relation ⌊Beziehung⌋ zwischen Zeit und Längen ⌊Raum⌋ mit Rücksicht auf die Fortpflanzungsgeschwindigkeit des Lichtes im leeren Raume geschaffen, und i ⌊behauptet. I⌋ndem ich auf die Eulersche Entdeckung vom Zusammenhang der trigonometrischen Funktionen und der Logarithmen Bezug nehme, ⌊eine Entdeckung⌋ welche seit ⌊nahezu⌋ zwei Jahrhunderten die Entwicklung der Mathematik beherrscht, so ⌊könnte kann ich man sagen, es⌋ verhält sich nach dem neuen Relativitätsprinzip die Zeit ⌊etwa ungefähr⌋ 1/30'000 von einem milliontel einer Sekunde ⌊und⌋ 1 cm wie zwei rechte Winkel zu der Ludolphschen Zahl ⌊durch eine imaginäre Drehung um einen imaginären Winkel zur Deckung gebracht werden können⌋. Ich verlange nicht, dass dieses so begriffen werde, aber wenn ich Sie versichere, dass dieser Behauptung ganz bestimmte und höchst subtile experimentelle Tatsachen einen sehr vernünftigen Sinn verschaffen, so werden Sie jedenfalls ⌊vielleicht⌋ überzeugt sein, dass es mit dem Relativitätsprinzip eine höchst merkwürdige Bewandtniss hat. Genug, dieses Prinzip kommt in einem ganz bestimmten Sinne zu dem Resultate ⌊Schlusse⌋, dass dem Zustande der Ruhe gegenüber der Bewegung keinerlei Eigenschaften der Erscheinungen entsprechen.

Ich habe mir nun die Frage vorgelegt, ob denn die LORENTZsche Elektrodynamik bewegter Körper, aus welcher sich schliesslich ⌊allmählich nach und nach⌋ das Relativitätsprinzip herausentwickelt hat, wirklich ⌊tatsächlich⌋ mit der Forderung des Relativitätsprinzips im Einklang steht und dabei ⌊ich⌋ bin ich ⌊dabei⌋ zu einem nicht erwarteten Resultate gelangt. Es ist das ⌊jenes das⌋ keineswegs allgemein der Fall, es ist aber bis zu einem gewissen Grade der Fall, nämlich für nicht magnetisierte Körper, hier aber eigentlich auch nur durch einen reinen Zufall, nämlich weil Lorentz die Bedingung des Nichtvorhandenseins von Magnetismus in einer solchen Weise eingeführt hat, die ihrerseits durchaus nicht dem Relativitätsprinzipe entspricht, also gewissermassen durch eine zufällige Kompensation von zwei Widersprüchen gegen das Relativitätsprinzip. Weiter sind auch die Beziehungen, welche Lorentz zwischen elektrischem Strom und elektromotorischer Kraft, andererseits zwischen dielektrischer Erregung und elektromotorischer Kraft setzt, nur approximativ mit dem Relativitätsprinzip im Einklang. Ich habe nun selbst dasjenige System von Gleichungen für die Elektrodynamik bewegter Körper aufgestellt, welches in allen Hinsichten dem Relativitätsprinzipe gerecht wird, und ich bringe Gründe dafür bei, dass in der Tat damit die wahren Grundlagen für die Elektrodynamik bewegter Körper getroffen sein mögen.

Meine gegenwärtige Darstellung wendet sich in erster Linie an die Physiker, und ich habe deshalb den mathematischen Apparat auf ein Minimum beschränkt. Ein zweiter Aufsatz, den ich nach den Ferien vorlegen will, wird mehr mathematische Beiträge zur Elektrodynamik bringen.

## Conflict of Interest

The author declares no conflict of interest.

## Footnotes

1
Walter 2008.

2
Walter 2007; Petkov 2010.

3
Minkowski 1908.

4
Minkowski 1909. Reprinted and translated in Petkov 2010, on xiv–xlii.

5
Galison 1979, on 120, in his survey of Minkowski's manuscript notes on space and time already identified these pages as early versions of the introduction: “Of interest is what seems to be a version of the introduction to this paper.”

6
Haffner and Bustamente 2025.

7
Walter 1999.

8
Schwermer 1991.

9 See the online member directory of the Göttingen Academy of Sciences and Humanities in Lower Saxony, https://adw‐goe.de/en/members. Minkowski was elected corresponding member (as a resident of Zurich) in the session of October 26, 1901, see “Bericht des vorsitzenden Sekretärs” 1902, on 82.

10
Minkowski 1897, 1899, 1900.

11
Minkowski 1904.

12
“Verzeichnis der im Jahre 1905/6 abgehaltenen Sitzungen” 1906, on 6.

13
Weber 1907.

14 David Hilbert, e.g., had been appointed privy council in 1904 after declining a call to the University of Heidelberg, see Sauer 2000, on 189. The appointment letter is an impressive document of Royal pomp in particular with its proverbial ornamental signature: “Wir Wilhelm, von Gottes Gnaden König von Preußen etc: thun kund und fügen hiermit zu wissen, daß Wir Allergnädigst geruht haben, den ordentlichen Professor in der Philosophischen Fakultät der Universität in Göttingen Dr. David Hilbert den Charakter als Geheimer Regierungsrath zu verleihen. Es ist dies in dem Vertrauen geschehen, daß derselbe Uns und Unserem Königlichen Hause in unverbrüchlicher Treue ergeben bleiben und seine Amtspflichten mit stets regem Eifer erfüllen werde, wogegen derselbe sich Unseres Allerhöchsten Schutzes bei den mit seinem gegenwärtigen Charakter verbundenen Rechten zu erfreuen haben soll. Urkundlich haben Wir dieses Patent AllerhöchstSelbst vollzogen und mit Unserm Königlichen Insiegel versehen lassen. Gegeben Neues Palais, den 14. November 1904. Wilhelm.” Staats‐ und Universitätsbibliothek Göttingen (= SUB) Cod. Ms. Hilbert 743/7.

15 Another exception was the 78‐year‐old comparative linguist Leo Meyer (1830–1910). Meyer had obtained both his doctorate and his habilitation (1856) in Göttingen and had then been appointed extraordinary professor in 1862 before accepting a chair for comparative linguistics at the University of Dorpat in 1865. He had been elected corresponding member to the Göttingen Academy in that same year. In 1899, he left the German university at Dorpat in the course of its russification and returned to Göttingen where he was then appointed honorary professor.

16
Volkert 2023.

17
Hilbert 1900, 1901.

18
Corry 1997.

19
Pyenson 1979.

20
Einstein 1905. Reprinted with historical annotation in Stachel 1989, Document 23.

21
Klein et al. 1993, Document 62.

22
Lorey 1916; Tobies 2019, Section 7.2.

23
Minkowski 1915, 1916.

24
“Mitteilungen und Nachrichten” 1908, on 4–5. All translations are by the author.

25 Ibid., on 6.

26 SUB Math.Arch.60.4. Both versions are contained in an envelope with cover remark “Ansprache in d. Ges. d. Wiss. bei Vorlage der ‘Grundgleichungen.’” The manuscript version, written in ink, consists of four pages, sequentially numbered in the upper right corners 1 to 4, the typescript version is actually a carbon copy of a typewritten text, consisting of five pages, with typed page numbers 1 to 5 centered at the top of the page. It displays a number of handwritten corrections in pencil in Minkowski's hand. The appendix gives a faithful transcription of both drafts, including all deletions and ⌊interlineations⌋.

27
Hertz 1890a.

28
Hertz 1890b.

29
Hertz 1892.

30
Ebel 1962.

31 “so ist nach dem neuen Relativitätsprinzip 1 cm zu der Zeit 1/30000 von einem millionsten einer Sekunde wie 2 rechte Winkel zu der Ludolphschen Zahl *π*.”

32
Minkowski 1910.

33
Walter 1999.

34
Minkowski 1909.

35 Sauer, forthcoming.

36 SUB Math.Arch.60.4, see note 26 above. At the top of the facing left hand page, Minkowski wrote: “Konink.Akad.v.Wetensch.//te Amsterdam 31. März 1900.” This is probably a reference to Lorentz's paper, “Considerations on Gravitation,” presented at the session of March 31, 1900, see Lorentz 1900.

## References

[bewi70018-bib-0001] “Bericht des Vorsitzenden Sekretärs über das hundertundfünfzigjährige Jubiläum der Gesellschaft,” Nachrichten von der Königl. Gesellschaft der Wissenschaften zu Göttingen. Geschäftliche Mitteilungen aus dem Jahre 1901 (1902): 79–130.

[bewi70018-bib-0002] Corry, Leo , “Hermann Minkowski and the Postulate of Relativity,” Archive for History of Exact Sciences 51 (1997): 273–314.

[bewi70018-bib-0003] Ebel, Wilhelm , Catalogus Professorum Gottingensium 1734–1962 (Göttingen: Vandenhoeck & Ruprecht, 1962).

[bewi70018-bib-0004] Einstein, Albert , “Zur Elektrodynamik bewegter Körper,” Annalen der Physik 17, no. 891 (1905): 891–921.

[bewi70018-bib-0005] Galison, Peter , “Minkowski's Space‐Time: From Visual Thinking to the Absolute World,” Historical Studies in the Physical Sciences 10 (1979): 85–121.

[bewi70018-bib-0006] Haffner, Emmylou , and Martha Cecilia Bustamente , “Dans l'atelier des sciences exactes,” Genesis 60 (2025): 7–17.

[bewi70018-bib-0007] Hertz, Heinrich , “Ueber die Grundgleichungen der Electrodynamik für ruhende Körper,” Annalen der Physik und Chemie 40, no. 8 (1890a): 577–624.

[bewi70018-bib-0008] Hertz, Heinrich , “Ueber die Grundgleichungen der Electrodynamik für ruhende Körper,” Nachrichten von der Königl. Gesellschaft der Wissenschaften zu Göttingen. Mathematisch‐physikalische Klasse (1890b): 106–149.

[bewi70018-bib-0009] Hertz, Heinrich , “Ueber die Grundgleichungen der Elektrodynamik für ruhende Körper,” in Heinrich Hertz, Untersuchungen über die Ausbreitung der elektrischen Kraft (Leipzig: Barth, 1892), 208–255.

[bewi70018-bib-0010] Hilbert, David , “Mathematische Probleme: Vortrag, gehalten auf dem internationalen Mathematiker‐Kongreß zu Paris 1900,” Nachrichten von der Königl. Gesellschaft der Wissenschaften zu Göttingen. Mathematisch‐physikalische Klasse (1900): 253–297.

[bewi70018-bib-0011] Hilbert, David , “Mathematische Probleme,” Archiv für Mathematik und Physik 1 (1901): 44–63, 213–237.

[bewi70018-bib-0012] Klein, Martin J. , Anne J. Kox , and Robert Schulmann (eds.), The Collected Papers of Albert Einstein. Volume 5: The Swiss Years: Correspondence, 1902–1914 (Princeton, NJ: Princeton University Press, 1993).

[bewi70018-bib-0013] Lorentz, Hendrik Antoon , “Considerations on Gravitation,” Koninklijke Akademie van Wetenschappen te Amsterdam: Proceedings of the Section of Sciences 2 (1900): 559–574.

[bewi70018-bib-0014] Lorey, Wilhelm , Das Studium der Mathematik an den deutschen Universitäten seit Anfang des 19. Jahrhunderts (Leipzig and Berlin: Teubner, 1916).

[bewi70018-bib-0015] Minkowski, Hermann , “Allgemeine Lehrsätze über die convexen Polyeder,” Nachrichten von der Königl. Gesellschaft der Wissenschaften zu Göttingen. Mathematisch‐physische Klasse (1897): 198–219.

[bewi70018-bib-0016] Minkowski, Hermann , “Ein Kriterium für die algebraischen Zahlen,” Nachrichten von der Königl. Gesellschaft der Wissenschaften zu Göttingen. Mathematisch‐physikalische Klasse (1899): 64–88.

[bewi70018-bib-0017] Minkowski, Hermann , “Zur Theorie der Einheiten in den algebraischen Zahlkörpern,” Nachrichten von der Königl. Gesellschaft der Wissenschaften zu Göttingen. Mathematisch‐physikalische Klasse (1900): 90–93.

[bewi70018-bib-0018] Minkowski, Hermann , “Dichteste gitterförmige Lagerung kongruenter Körper,” Nachrichten von der Königl. Gesellschaft der Wissenschaften zu Göttingen. Mathematisch‐physikalische Klasse (1904): 311–355.

[bewi70018-bib-0019] Minkowski, Hermann , “Die Grundgleichungen für die elektromagnetischen Vorgänge in bewegten Körpern,” Nachrichten von der Königl. Gesellschaft der Wissenschaften zu Göttingen. Mathematisch‐physikalische Klasse (1908): 53–111.

[bewi70018-bib-0020] Minkowski, Hermann , “Raum und Zeit,” Physikalische Zeitschrift 10 (1909): 104–111.

[bewi70018-bib-0021] Minkowski, Hermann , “Eine Ableitung der Grundgleichungen für die elektromagnetischen Vorgänge in bewegten Körpern. Aus dem Nachlaß von Hermann Minkowski bearbeitet von Max Born in Göttingen,” Mathematische Annalen 68 (1910): 526–551.

[bewi70018-bib-0022] Minkowski, Hermann , “Das Relativitätsprinzip,” Annalen der Physik 47 (1915): 927–938.

[bewi70018-bib-0023] Minkowski, Hermann , “Das Relativitätsprinzip,” Jahresbericht der Deutschen Mathematiker‐Vereinigung 24 (1916): 372–382.

[bewi70018-bib-0024] “Mitteilungen und Nachrichten,” Jahresbericht der Deutschen Mathematiker‐Vereinigung 17 (1908): 1–12.

[bewi70018-bib-0025] Petkov, Vesselin (ed.), Minkowski Spacetime: A Hundred Years Later (Dordrecht: Springer, 2010).

[bewi70018-bib-0026] Pyenson, Lewis , “Physics in the Shadow of Mathematics: The Göttingen Electron‐Theory Seminar of 1905,” Archive for History of Exact Sciences 21, no. 1 (1979): 55–89.

[bewi70018-bib-0027] Sauer, Tilman , “Hilberts Ruf nach Bern,” Gesnerus 57 (2000): 182–205.

[bewi70018-bib-0028] Schwermer, Joachim , “Räumliche Anschauung und Minima positiv definiter quadratischer Formen: Zur Habilitation von Hermann Minkowski 1887 in Bonn,” Jahresbericht der Deutschen Mathematiker‐Vereinigung 93, no. 2 (1991): 49–105.

[bewi70018-bib-0029] Stachel, John (ed.), The Collected Papers of Albert Einstein. Volume 2: The Swiss Years: Writings, 1900–1909 (Princeton, NJ: Princeton University Press, 1989).

[bewi70018-bib-0030] Tobies, Renate , Felix Klein: Visionen für Mathematik, Anwendungen und Unterricht (Heidelberg: Springer Spektrum, 2019).

[bewi70018-bib-0031] “Verzeichnis der im Jahre 1905/6 abgehaltenen Sitzungen und der darin gemachten wissenschaftlichen Mittheilungen,” Nachrichten von der Königl. Gesellschaft der Wissenschaften zu Göttingen. Geschäftliche Mittheilungen 1906: Heft 1 (1906): 5–10.

[bewi70018-bib-0032] Volkert, Klaus , “Drei mathematische Freunde über Poincaré,” Philosophia Scientiae 27, no. 2 (2023): 129–157.

[bewi70018-bib-0033] Walter, Scott , “Minkowski, Mathematicians and the Mathematical Theory of Relativity,” in The Expanding Worlds of General Relativity, ed. Hubert Goenner , Jürgen Renn , Jim Ritter , and Tilman Sauer (Boston and Basel: Birkhäuser, 1999), 45–86.

[bewi70018-bib-0034] Walter, Scott , “Breaking in the 4‐Vectors: The Four‐Dimensional Movement in Gravitation, 1905–1910,” The Genesis of General Realtivity, vol. 3, ed. Jürgen Renn (4 vols., Berlin: Springer, 2007), 193–252.

[bewi70018-bib-0035] Walter, Scott , “Hermann Minkowski's Approach to Physics,” Mathematische Semesterberichte 55, no. 2 (2008): 213–235.

[bewi70018-bib-0036] Weber, Heinrich , “Über die Komposition der quadratischen Formen,” Nachrichten von der Königlichen Gesellschaft der Wissenschaften zu Göttingen. Mathematisch‐physikalische Klasse (1907): 86–100.

